# Territorial behaviour of thrush nightingales outside the breeding season

**DOI:** 10.1098/rspb.2023.0496

**Published:** 2023-08-30

**Authors:** Henrik Brumm, Léna de Framond, Wolfgang Goymann

**Affiliations:** ^1^ Communication and Social Behaviour Group, Max Planck Institute for Ornithology, 82319 Seewiesen, Germany; ^2^ Department of Behavioural Neurobiology, Max Planck Institute for Ornithology, 82319 Seewiesen, Germany

**Keywords:** animal communication, bird song, *Luscinia luscinia*, song amplitude, song development, territoriality

## Abstract

Territoriality is a common pattern of space use in animals that has fundamental consequences for ecological processes. In the tropics, all-year resident songbirds usually hold territories throughout the year, whereas most all-year resident temperate species are territorial only during the breeding season. In long-distance migrants, however, the situation is mostly unexplored. Here, we report findings from a Palaearctic–African migrant, the thrush nightingale *Luscinia luscina*. We found that only a fraction of the males was territorial in their East African winter quarters and that this was related to the stage of their song development. Individuals with full song were territorial towards other full songsters, but not towards birds that sang plastic song (i.e. an earlier stage of song development). Plastic singers were not territorial towards full songsters and often settled closely to territorial males. We suggest that territoriality of thrush nightingales in the winter quarters may be a by-product of rising testosterone levels that trigger song crystallization. Collectively, our study indicates that changes in territoriality can occur rapidly, giving rise to shifting proportions of territorial and non-territorial individuals in a population, which may lead to complex dynamics in settlement patterns and resulting ecological interactions.

## 1. Introduction

Animals compete for resources, such as food, breeding sites, shelters, and mates. Often they vie by keeping others away from the area containing the resources. This behavioural strategy is known as ‘territoriality’ and the defended area as the ‘territory’ [1]. Territoriality is a fundamental concept in animal behaviour and behavioural ecology [2–4] as it has consequences for reproductive success [5] as well as many critical ecological processes, such as predator–prey interactions [6], disease transmission [7], community structure [8,9] and population density and dynamics [10,11].

In addition to physical aggression, territoriality can be mediated by the production of special advertisement signals that are used to exclude competitors from certain areas. Territorial frogs, for instance, use loud calls to keep rivals at bay [12], whereas Anolis lizards defend territories with visual displays [13]. Antelopes, just like many other mammals, flag their territory boundaries with scent marks [14] and certain caterpillars have been found to produce vibratory signals to repel conspecifics from their shelters [15].

In perching birds, territories are established and maintained by advertisement songs (reviewed by [16]). Oscines acquire their songs through vocal production learning [17] and the ontogenetic development of their singing follows a typical route from subsong to plastic song and then to crystallized full song [18]. Subsong consists of soft and highly variable vocalizations that do not contain memorized song patterns. The emergence of memorized song material ushers the plastic song phase, during which note structure and song syntax are elaborated. This may take several weeks to months, depending on the species. In a last step, the ‘song crystallization’, birds advance to their stereotyped full song. In addition to the structural differences, full song is usually also markedly louder than plastic song [19]. In many temperate bird species, males recap this ontogenetic development each year and go through a phase of plastic singing before their song takes on its full form again in the breeding season [20–22].

In long-distance migrants, the plastic song phase normally happens while the birds are in their winter quarters and it has been hypothesized that they may learn new songs during this period [23,24]. One of the first to suggest such song learning in tropical winter quarters was the ornithologist Françoise Dowsett-Lemaire, who noticed potential imitations of vocalizations from African resident species in the songs of marsh warblers (*Acrocephalus palustris*) recorded in Europe [25]. In line with the song learning function, plastic singing is usually regarded as motor practice [26–28], but it is unknown whether plastic song may also be used for territorial functions.

Life histories often vary between the tropics and the temperate regions, and this also seems to be true for the duration of territory maintenance in songbirds: in the tropics, all-year resident species are usually territorial all year round, whereas the majority of all-year resident temperate species are not [29]. Yet in long-distance migrants, which breed in the temperate zones and spend the non-breeding season in the tropics, the situation is still largely unresolved. Many Palaearctic–African migratory songbirds sing on their wintering grounds (e.g. [24,30–33]), but song alone cannot be taken as an indicator of territoriality because it may have other functions outside the breeding season [34]. Moreover, it remains unclear whether plastic song, which usually occurs during the wintering stage, is used for territorial functions at all. What is lacking are rigorous tests of territorial behaviours. Such testing could involve the spacing of males [35] or physical aggression towards competitors [36] or the birds' responses to song playback [37]. Notable studies that did investigate potential territoriality of Palaearctic–African migrants in their winter quarters are those by Sorensen and colleagues, who found that willow warblers (*Phylloscopus trochilus*) defend only short-term territories in their tropical winter quarters [32] and that great reed warblers (*Acrocephalus arundinaceus*) are not territorial on their wintering grounds despite frequent singing activity [34].

We studied territorial behaviour on the wintering grounds of a passerine Palaearctic–African migrant, the thrush nightingale (*Luscinia luscinia*). When we were recording bird songs in the Usangu plains in Southern Tanzania [38], we noticed two thrush nightingales singing for extended periods from the same thicket: one was singing full song, while the other produced plastic song that sounded softer and lacked the typical structure of the advertisement song of thrush nightingales. The short distance of only 15 m between the two singing males was striking because song perches in the breeding area are usually spaced at least 50–100 m apart [39]. Hence, we wondered whether the birds are at all territorial on their wintering grounds and if so, whether territoriality depends on the song stage of the involved individuals. The organization of thrush nightingale song and its use in the defence of breeding territories has been well studied (e.g. [40–43]). Territorial aggression in this species typically involves approaching rival males and counter-singing [44,45]. When territorial interactions escalate, chases and physical fights can be observed. In their East African winter quarters [46], thrush nightingales typically sing between December and March [47], with many birds producing plastic song [24]. Here we report the findings of a 5-year study on the behaviour of thrush nightingales on their wintering grounds. To investigate the role of song ontogeny in territorial behaviour, we quantified the spacing of singing males in relation to their song stage and conducted playback experiments to test the birds’ responses to plastic and full song.

## 2. Methods

**Figure 1.**
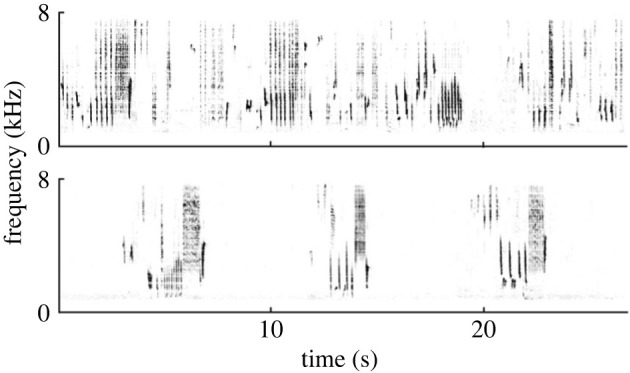
Spectrograms of typical thrush nightingale song from the winter quarters in Tanzania. Top panel: plastic song, bottom panel: full song. While full song is discontinuous (i.e. short strophes separated by silent intervals), plastic song has a more continuous form.

In 2016, 2018, 2019, 2021 and 2022 we opportunistically surveyed areas in Tanzania between latitude −2°15′22.3 and −8°55′51.4, targeting suitable thrush nightingale habitat. The exact observation periods differed between years, they lasted between 19 and 30 days and laid between 13 February and 17 April. During this time, we observed 87 singing birds and noted whether they were producing plastic or full song (figure 1). In full song, strophes typically have a duration of 4.4–6.4 s [48] and are separated by pauses between 1.0–7.0 s (de Framond & Brumm 2023, unpublished data). Plastic song, on the other hand, is distinguished by longer, continuous song bouts (figure 1), and more variable syllables [24]. As an operational definition, we scored songs with bout durations longer than 7 s as plastic. Usually, plastic song also sounds softer than full song and has a more rambling and restrained tone.

In addition to the large-scale survey, we explored territorial behaviour in more detail in the Usangu plains in southwestern Tanzania, where we measured the spacing between males and conducted playback experiments. For the exact location of our field site and detailed descriptions of the local savannah habitat see [49,50]. To quantify male spacing, we thoroughly surveyed the area on foot and on bicycle in the morning hours and took GPS coordinates of the song perches of all birds that we heard singing. The birds at our field site appeared to be stationary during the annual observation period of two to three weeks, as we typically heard song from the same locations every morning until the birds left for spring migration in late March. The thrush nightingales settled almost always in thickets (e.g. *Acacia* sp.) and the rather open habitat with scattered bushes and solitary trees interspersed with open areas made it easy to detect and map the birds' locations.

For the design of realistic playback experiments, it is vital to consider the source levels of the respective signals [51,52]. Therefore, we measured the amplitude of thrush nightingale song on their wintering grounds. This was done in 2016 and 2018 according to established protocols [38,53]. In brief, we made calibrated recordings of singing thrush nightingales from a close distance (14–21 m, measured with a Leica Rangemaster 800 laser range finder) with a Sennheiser ME 66 microphone (with a Sennheiser MZW 66-PRO windshield) connected to a Marantz PMD 660 solid-state recorder (44.1 kHz, 16 bit). The recording equipment was calibrated in an anechoic room (see [54] for details) a few days before the beginning of the field season and we also recorded reference tones regularly over the course of the field work. Yet the initial calibration proved to be sufficient as the reference tones recorded in the field deviated only very little from the calibrated value (1.5 dB or less). The calibrated recordings allowed us to measure the standardized song amplitude [53], and the mean values of plastic and full song were then used to set amplitudes for the playback experiment.

### (a) Playback experiments

Playback experiments were conducted during 10–24 March 2018, 26 February–1 March 2019, on 1 April 2021, and during 11–29 March 2022. We carried out the experiments between 07.00 and 12.50 on mornings with no rain. Altogether, we tested 41 males (22 plastic songsters and 19 full songsters) with a full song playback. Thirteen of the full songsters also received a plastic song playback. The order of the two playback types was balanced between subjects (as much as it is possible in an uneven number). In the paired design, each bird received both playbacks on the same day with a break of 30 min in between them.

The source recordings for the playback files came from birds that we recorded in Tanzania with a Marantz PMD 660 solid-state recorder (44.1 kHz, 16 bit) and a Sennheiser ME 66 microphone (either with a MZW 66-PRO windshield or a RØDE Blimp windshield and shock-mount system). From each source bird we chose 1 min of song that we high-pass filtered (1024-tap finite impulse response, cut-off frequency 0.9 kHz, Hamming window) with the software Avisoft SASLab Pro (v. 5.3.01, Avisoft GmbH, Berlin, Germany) to remove low-frequency noise outside the frequency band of thrush nightingale song. The filtered 1-min section was then used as a playback file. In total, we had 12 source males for full songs and 17 for plastic songs. Each subject received a playback that had been recorded in a previous year at a location between 1 and 150 km away.

At the beginning of a playback trial, subjects were localized by their song and the loudspeaker of a remote-controlled playback device (Foxpro Scorpion) was set up 20 m from the focal bird in a bush about 1.6 m above the ground and facing towards the focal bird. The playback volume was set according to the measured mean amplitudes of plastic and full song (see results). After the focal bird remained silent for at least 30 s we started the playback and recorded the subject's behaviour during the playback and a 2-min post-playback period (which were combined for the analysis). The closest approach to the playback loudspeaker was estimated by one observer in the field (H.B.) and the vocal responses of the birds were recorded with Sennheiser ME 66 microphone (mounted in a RØDE Blimp windshield and shock-mount system) that was connected to a Marantz PMD 660 recorder (44.1 kHz, 16 bit). The number of songs produced by each subject was determined from the audio recordings in a blinded procedure, i.e. the person counting the songs was not informed about the treatments (but the playback treatments were audible in the recordings).

### (b) Singing activity

Between 18 and 31 March 2022 we recorded diurnal patterns of singing activity in five selected males at our field site in the Usangu plain. For this, we used AudioMoth audio recorders (v. 1.2.0) [55] that we set up within 20 m from a given male's song perch to obtain continuous recordings for 24 h (sample rate 32 kHz, gain ‘medium’). Each male was recorded on a different day and their locations were at least 1 km apart. The recordings were visualized as spectrograms (512-point FFT) with Avisoft SASLab Pro and we analysed the birds singing activity using a one-zero sampling with a sample interval of 1 min [56].

### (c) Statistical analyses

All statistical analyses were performed in R (v. 4.2.1) under a Bayesian framework using the package *brms* (v. 2.17.6). The birds' responses to playback were investigated in relation to the three experimental treatments (full-song singers receiving full-song playback, full-song singers receiving plastic song playback, and plastic-song singers receiving full song playback). For this, we used two different models, one for the number of songs and one for the approach distance. We modelled the number of songs in response to playback as a function of the treatment group using a GLMM from the Poisson family, with the treatment group as a categorical variable and the time of day as a linear variable (in hours after sunrise). The approach distance was modelled with a GLMM with an exponential error distribution, and with the treatment group as a categorical variable and the time of day as a linear variable (in hours after sunrise). In both models, we included bird identity as a random factor to account for repeated measures.

To investigate the spacing between males, we calculated a proximity index between each focal bird and his nearest neighbour of either song stage (formula 1). We only considered neighbours within a radius of 200 m around the focal birds (which is a greater distance than the typical spacing of neighbouring birds in the breeding range [39]). With this index, a value of 1 corresponds to a distance of 10 m to the nearest neighbour, and 0 corresponds to no neighbour at all (0 is a theoretical number though, in practice 0 was replaced by 10^−7^ because exponential GLMMs cannot model 0 values in *brms*).

Formula (1)

$$\begin{array}{rl} & \mathrm{Proximity}\;\mathrm{index}=\{\begin{array}{cc} \frac{10}{\mathrm{distance}\;\;\mathrm{to}\;\mathrm{neighbour}} & \mathrm{if}\;\mathrm{neighbour}\;\mathrm{present}\;\\ 10^{-7}\; & \mathrm{if}\;\mathrm{no}\;\mathrm{neighbour}\;\mathrm{present} \end{array} \end{array}$$

We modelled the proximity index to the nearest neighbour (of either song stage) as a function of the neighbours’ song stage. For this, we used a GLMM with an exponential error distribution and with the identity of each focal bird as a random effect to account for individual differences among birds. For all models, we used the default, non-informative flat improper priors [57] and four chains of 5000 iterations each, including a warm up of 2500 iterations and a thinning rate of 4. We performed posterior-predictive checks to assess the model fit and examined chain convergence using the visualization tools of the package *bayesplot* (v. 1.9.0) following [58]. Graphs of predicted values were made with the package *ggeffect* (v. 1.1.2). If not indicated otherwise, data are reported as posterior means and their 95% credible intervals.

## 3. Results

### (a) Song stages and singing activity

**Figure 2.**
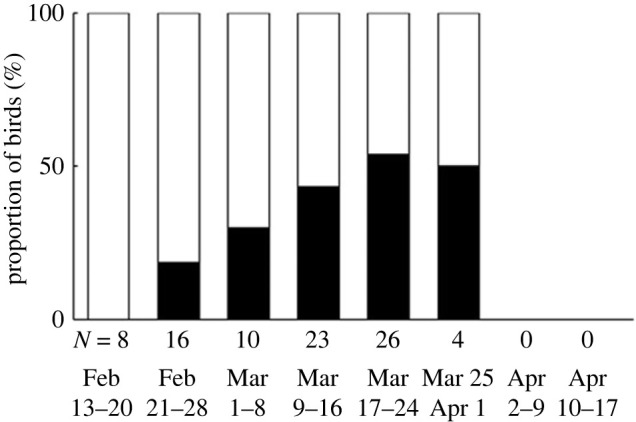
The proportion of full songsters increases during the course of the wintering stage. The proportions of plastic (white) and full songsters (black) are given in bins of one week. *N* denotes the number of observed males per bin. The latest record of a singing bird was 1 April.

The proportion of birds singing full songs increased from none in the third week of February to about 50% at the end of March after which birds departed on spring migration to their Palaearctic breeding grounds (figure 2).

With a median peak amplitude of 84.3 dB SPL at 1 m (range of individual medians: 83.9–87.2) full songs were markedly louder than plastic songs, which measured on average only 75.0 dB SPL at 1 m (range of individual medians: 69.7–78.1; electronic supplementary material, table S1). Our monitoring of the singing activity of selected males showed that the birds sang only during daylight hours (between 06.40 and 16.12 h) and never during the night (electronic supplementary material, figure S1). The majority of the song output was restricted to the morning, with peaks between 07.30 and 09.30 h. The monitored plastic songsters sang roughly three times as much as the full songsters, but in view of the small sample size of five birds we are unable to draw firm conclusions from this observed difference.

### (b) Distances between birds

**Figure 3.**
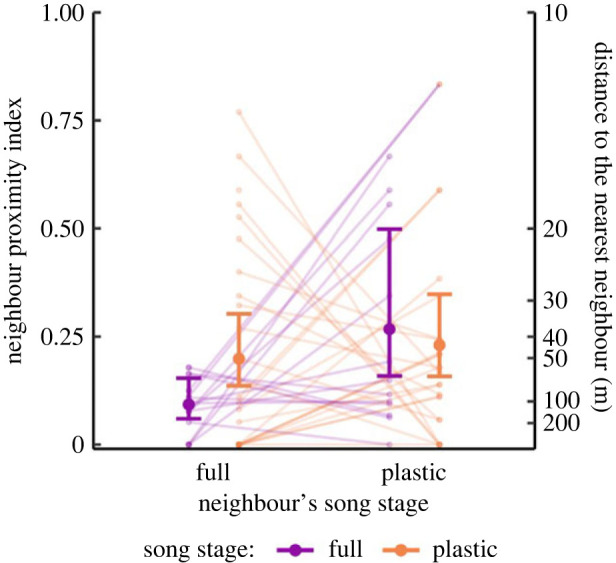
Full songsters keep a greater distance from other full songsters than from plastic singers. Proximity index (posterior means and 95% credible interval) between the song perches of full singers (purple) and plastic singers (orange) and their nearest full-song and plastic-song neighbours. Lines show the data for each individual bird (one line per bird, *N* = 46 males). A scale indicating the distance between neighbours in metres is shown on the right for context.

The average proximity index between full songsters was 0.09 [0.06, 0.15] (exponential GLMM estimate and 95% credible interval, transformed into linear scale), which corresponds to a distance of 107.4 m [65.0, 166.9]. The song stage of the focal bird, his neighbours' song stage, and their interaction all had an effect on the distance in which the males settled from each other (table 1). In comparison to the spacing between neighbouring full songster, the nearest neighbours of plastic singers were found at considerably closer distances, on average less than 50 m (figure 3). When considering the neighbours of full songsters, it is striking that many plastic songsters settled closer than 30 m from them (44% of all cases), whereas other full songsters were never observed at such a close range (figure 3).

**Table 1.** Exponential GLMM estimates (posterior means and 95% credible interval) of the proximity index between neighbours as a function of focal birds' song stage and neighbour's song stage. Model results of exponential models are on the log scale.

	estimate [95% CrI]
intercept (focal bird full song – neighbour full song)	−2.36 [−2.82, −1.87]
focal bird's song stage (plastic)	0.76 [0.11, 1.36]
neighbour's song stage (plastic)	1.06 [0.33, 1.85]
focal bird's song stage : neighbour's song stage (plastic-plastic)	−0.92 [−1.86, 0.00]

### (c) Responses to playback

**Figure 4.**
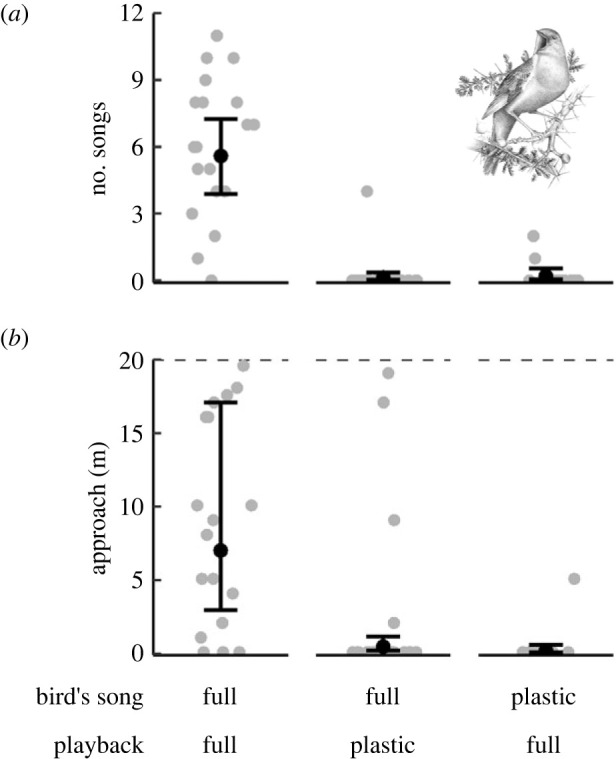
Full singers respond aggressively to other full singers, but not to plastic singers, and plastic singers do not respond aggressively to full singers either. (*a*) Number of songs and (*b*) approached distance in relation to the song stage of the focal bird (bird's song) and the playback type (playback). Grey dots are raw data points; black dots and whiskers denote posterior means and 95% credible intervals from the GLMMs. The dotted line denotes the maximum distance a bird could approach the loudspeaker (i.e. the distance between the loudspeaker and the bird at the start of the experiment). Illustration by L. de Framond.

Birds in the full song stage responded to broadcasts of full song by singing, on average, 5.42 songs (Poisson GLMM 95% CrI [3.68, 7.44], figure 4*a*) and by approaching the loudspeaker on average by 7.15 m (exponential GLMM 95% CrI translated into linear scale [3.01, 16.68], figure 4*b*). By contrast, they responded hardly at all to plastic song (table 2). On average, they sang only 0.1 songs (figure 4*a*) and approached the loudspeaker by 0.1 m (figure 4*b*). A similar lack of a response was observed in plastic songsters in response to full song broadcasts (figure 4*a,b*, table 2). The time of day did not affect the birds’ reactions to the playback (table 2). These findings were confirmed by a sensitivity analysis on a subset of the data: we cannot rule out that the same individuals might have unintentionally been re-sampled in different years, we repeated the analysis with only the birds tested in 2022, which yielded basically the same result (electronic supplementary material, table S2).

**Table 2.** Birds' response (GLMM posterior mean estimate and 95% credible intervals) to full song and plastic song playbacks. The number of songs (a) and minimum approach to the loudspeaker (b) were modelled with a Poisson (a) and an exponential (b) GLMM. Note that the estimates are on the log scale.

	(a) number of songs	(b) approach
	estimate [95% CrI]	estimate [95% CrI]
intercept (bird full song – playback full song)	1.69 [1.30, 2.01]	1.97 [1.10, 2.81]
bird full song – playback plastic song	−3.40 [−4.88, −2.29]	−3.50 [−4.46, −2.54]
bird plastic song – playback full song	−3.79 [−5.17, −2.76]	−2.76 [−3.95, −1.52]
time of day (hours after sunrise)	0.00 [−0.30, 0.27]	0.27 [−0.22, 0.74]

## 4. Discussion

Our study of thrush nightingales in their East African non-breeding range showed that the spacing between males and their responses to simulated conspecifics varied with their song development stage. Birds with full song responded with typical territorial behaviour to full song, but not to plastic song, and plastic songsters did not respond to full song. Full songsters chose their song posts on average 107 m from each other, which is similar to the spacing of breeding territories in Europe [39]. By contrast, plastic songsters settled considerably closer to full songsters, often below 30 m. Thus, we conclude (1) that thrush nightingales are territorial on their wintering grounds only after their songs have crystallized and (2) that they defend territories only against other full songsters, but are tolerant to plastic singers.

A proximate mechanism that links song crystallization with territorial aggression is the behavioural modulations mediated by steroid hormones. In particular, high testosterone levels have been found to promote song crystallization [59–61] as well as the intensity of territorial behaviours [62]. Testis size and testosterone concentrations of male long-distance migrants begin to increase already before or during migration [63–65]. In American redstarts (*Setophaga ruticilla*), for instance, testosterone levels rise in late winter, and they do so to a greater extent in older than younger males [66]. Similar age differences in testosterone levels could possibly be accounted for the observed differences in the timing of song crystallization in thrush nightingales in their East African winter quarters. Typical winter territoriality of migratory birds is regulated by different mechanisms depending on the species, with testosterone being involved in some [67] but not in other cases [36,68–70]. When testosterone concentrations begin to rise at the end of the wintering season, long-distance migrants start transitioning from the non-breeding to the breeding life-history stage [71,72]. We thus suggest that rising testosterone levels in preparation for the breeding season lead to song crystallization in thrush nightingales and, concomitantly, make them become territorial, even when they are still on their wintering grounds.

Our observation of plastic singers settling close to males with full song is reminiscent of juvenile song sparrows (*Melospiza melodia*) that approach singing adults, presumably to learn new songs [73]. Thrush nightingales are most likely open-ended learners [41,42] and hence they can probably learn new songs during the plastic song stage, just as it is the case with their sister species, the common nightingale *Luscinia megarhynchos* [74]. Although our data indicate that plastic singers are not preferentially attracted by full singers (they also aggregated at similar distances with plastic singers), the close distance to full singers may provide them with learning opportunities for new song types. Alternatively, plastic singers may be aggregating due to resource distribution (e.g. distribution of suitable thickets), not due to song learning, and they remain in the plastic singing stage to be tolerated in the patch that they settled in.

Territorial aggression in songbirds is modulated, among other things, by the amplitude of the rival's song [75] inasmuch as territorial males respond less aggressively to softer songs [76,77]. We found that plastic song in thrush nightingales is on average 9.4 dB softer than their full song, and this low amplitude may help plastic singers being tolerated by territorial males. Moreover, since plastic song in passerines appears to be generally softer than their full song [19,78,79], the phenomenon of differential territorial aggression, and the connected potential utilization of learning opportunities, might be much more widespread.

It is unclear, however, what the function of the winter territoriality in thrush nightingales is, especially because it only set in during the last five weeks before spring migration. We cannot rule out that full songsters defend feeding territories, but in this case one would expect them to direct territorial aggression towards any conspecific male and not only towards other full songsters. Moreover, territoriality does not seem to be a key strategy for thrush nightingales in their non-breeding range, as the majority of males are not territorial during most of the time. We suggest that territorial behaviour in the non-breeding season is a by-product of rising testosterone concentrations necessary for song crystallization. In other words, males that complete their song development already in their winter quarters happen to become territorial there, although territoriality may be adaptive in their breeding range only. However, the question whether territoriality is adaptive in the wintering area must, at this stage, remain open. It is possible, for instance, that territoriality in the winter quarters provides practice with this important behaviour ahead of the breeding season when errors in assessing rivals or producing signals are costlier.

In the breeding area, male thrush nightingales are strictly territorial, exhibiting strong antagonistic responses towards rivals, which typically results in spacing of territories of 50–100 m or more [39]. In addition, thrush nightingales sing during the night in the breeding season [44,80]. This behaviour is thought to be related to the other main function of bird song, i.e. mate attraction (c.f. [81]). The notion of nocturnal song as a mate-attraction signal is supported by our observation that males did not sing during the night in the non-breeding area, where mate attraction is not required. Thus, we may conclude that the emergence of nocturnal song and territoriality are triggered by different processes and that the mate-attraction function and the territorial function of song can arise independently from each other.

Our finding that the proportion between territorial and non-territorial birds shifted in the winter quarters has implications for settling patterns, as birds will move further away from full songsters once they reach the full song stage themselves. Because of this, settling patterns in thrush nightingales, and probably other species too, are dynamic and with predictable shifts during the course of the non-breeding season. When the birds left our field site for spring migration, about half of the population was still non-territorial (i.e. in the plastic song stage). This divergence in territoriality may be accounted for by differences in the birds' age (see above). Our non-invasive approach did not allow us to establish the age of the observed birds, however. Territoriality and age can be linked because song development in first-year males is often slower than the ontogenetic recapitulation of the process in older birds, resulting in older males changing over to full song earlier than young ones [82]. Moreover, older birds may respond more strongly to conspecific playback than yearling males in the wintering areas [83]. Accordingly, thrush nightingales that become territorial on the wintering grounds are perhaps older birds, whereas first-year birds remain in plastic song, and hence non-territorial, until their departure to the breeding areas. If this is the case, the territorial responses of the thrush nightingales on their wintering grounds would be similar to resident tropical rufous-collared sparrows (*Zonotrichia capensis*) that seem to be generally tolerant of juveniles [84]. In resident temperate song sparrows (*Melospiza melodia*), however, territorial males have been found to tolerate juveniles only in summer but not in early spring when juveniles sing late plastic song [85]. The key difference here may be that plastic song in temperate resident birds is produced in the breeding area, whereas in temperate long-distance migrants it coincides with their sojourn in the wintering area. Therefore, it is probably not the migratory strategy *per se* that determines territorial aggression towards plastic song but rather whether or not the song is produced in the breeding area.

In summary, we found that thrush nightingale song is not necessarily indicative of territoriality in the non-breeding range. Only males in the full stage were territorial on their wintering grounds, but plastic singers were not. It is tempting to imagine that song crystallization requires rising levels of testosterone and that territorial behaviour may arise as a non-adaptive side effect of song maturation. Further studies with individually identified birds and sampling of hormone concentrations are required to test this idea. It is known that animals become territorial in certain seasons [86] or life-history stages [87]. Beyond that, our study indicates that the proportions of territorial and non-territorial individuals in a population may shift rapidly, which may lead to complex dynamics in the resulting settlement patterns.

## Data Availability

The data used in this study are available from the Open Research Data Repository of the Max Planck Society (https://doi.org/10.17617/3.7KF4JR) [88] and in the electronic supplementary material [89].
